# An Open-Source, Interoperable Architecture for Generating Real-Time Surgical Team Cognitive Alerts from Heart-Rate Variability Monitoring

**DOI:** 10.3390/s23083890

**Published:** 2023-04-11

**Authors:** David Arney, Yi Zhang, Lauren R. Kennedy-Metz, Roger D. Dias, Julian M. Goldman, Marco A. Zenati

**Affiliations:** 1 Medical Device Plug-and-Play Interoperability and Cybersecurity Program, Massachusetts General Hospital, Boston, MA 02115, USA; 2 Department of Anaesthesia, Harvard Medical School, Boston, MA 02115, USA; 3 Department of Psychology, Roanoke College, Salem, VA 24153, USA; 4 STRATUS Center for Medical Simulation, Department of Emergency Medicine, Brigham and Women’s Hospital, Boston, MA 02115, USA; 5 Division of Cardiac Surgery, Veterans Affairs Boston Healthcare System, Department of Surgery, Brigham and Women’s Hospital, Harvard Medical School, Boston, MA 02115, USA

**Keywords:** surgical data science, interoperability, cognitive load, smart alarms, heart-rate variability

## Abstract

Clinical alarm and decision support systems that lack clinical context may create non-actionable nuisance alarms that are not clinically relevant and can cause distractions during the most difficult moments of a surgery. We present a novel, interoperable, real-time system for adding contextual awareness to clinical systems by monitoring the heart-rate variability (HRV) of clinical team members. We designed an architecture for real-time capture, analysis, and presentation of HRV data from multiple clinicians and implemented this architecture as an application and device interfaces on the open-source OpenICE interoperability platform. In this work, we extend OpenICE with new capabilities to support the needs of the context-aware OR including a modularized data pipeline for simultaneously processing real-time electrocardiographic (ECG) waveforms from multiple clinicians to create estimates of their individual cognitive load. The system is built with standardized interfaces that allow for free interchange of software and hardware components including sensor devices, ECG filtering and beat detection algorithms, HRV metric calculations, and individual and team alerts based on changes in metrics. By integrating contextual cues and team member state into a unified process model, we believe future clinical applications will be able to emulate some of these behaviors to provide context-aware information to improve the safety and quality of surgical interventions.

## 1. Introduction

Cardiac Surgery is one of the most complex and high-risk areas of medicine. Four teams (Surgery, Anesthesia, Perfusion, and Nursing), each comprised of 2–3 individuals, need to cooperate and interact with medical cyber-physical systems to achieve the desired cardiac intervention (e.g., repair a defective mitral valve) effectively, safely, and expeditiously [1]. Effective team coordination requires the management of interruptions and distractions as well as awareness of the context of the surgical procedure. Cognitive load, a measure of how mentally occupied or stressed a team member is at a particular time, provides a useful metric for understanding how team members’ attention and mental load varies through the stages of a surgical procedure.

A context-aware operating room (OR) is one in which patient data are integrated with information about surgical processes, medical devices, and clinician mental states in order to provide OR providers, in real time, with an overall status of the surgical process, alerts and guidance about current and anticipated hazardous situations. Thus, the real-time monitoring and alerting of the cognitive load of surgical team members during the procedure can be an important application of the context-aware OR.

To establish context-aware OR infrastructures and applications, the emerging field of surgical data science strives to leverage the data-rich environment in the OR through the capture, formatting, analysis, and modelling of data to ultimately improve surgical care [2]. Building a context-aware OR requires multidisciplinary collaboration to not only capture disparate data sources, but also integrate device data, clinician mental state monitoring, and process modelling and monitoring. These new intelligent spaces offer great potential to improve patient safety and outcomes as well as to build rich new data stores for better understanding and improving clinical processes.

Unfortunately, current technologies capturing and integrating important patient data from clinical environments, such as widely used anesthesia record systems and electronic health records (EHR), suffer from significant limitations in recording key information necessary for understanding clinical processes. These systems are not suitable for building real-time alarms and closed-loop control applications, or for supporting the context-aware OR vision described above. For example, there may be delays of up to several minutes from the time a medication administration or other data element is entered at the bedside until it appears in the EHR report from that patient and detailed information from patient monitors such as waveforms are usually not recorded.

In this paper, we propose a framework and describe a novel application that both makes use of, and adds additional contextual information to, a context-aware OR based on the OpenICE (Open Integrated Clinical Environment) medical device interoperability framework [3]. This effort builds on previous work on surgical process modeling, behavioral and cognitive engineering [4], and an offline dashboard for cognitive load analysis [5]. In particular, we apply the OpenICE platform to the surgical subspecialty of Cardiac Surgery in order to enable a ‘smarter’, or more context-aware, operating room by developing real-time processing, monitoring, and alerting based on measures of individual and overall team cognitive load. We call this adaptation COR-ICE for the Cardiac Operating Room Integrated Clinical Environment.

OpenICE is an open-source implementation of the AAMI (Association for the Advancement of Medical Instrumentation) 2700-1:2019 standard on the patient-centric integrated clinical environment (ICE) interoperability architecture [6]. It is a platform for medical device integration that supports rapid development of bedside clinical applications including data collection, smart alarms and data dashboards, and closed-loop control of devices. OpenICE software code is freely available online: https://www.openice.info (accessed on 10 January 2023).

In this work, we extend OpenICE with new capabilities to support the needs of the context-aware OR including a modularized data pipeline for simultaneously processing real-time electrocardiographic (ECG) waveforms from multiple clinicians to create estimates of cognitive load.

This real-time ECG pipeline leverages previous work [5] on developing a dashboard for offline analysis of cognitive load estimates derived from heart-rate variability (HRV) metrics as well as extensive related work on HRV metrics and ECG signal processing and analysis algorithms [7,8].

Unobtrusive physiological sensors and the accompanying algorithms applied to the waveforms generated by them [9] have contributed substantially to our understanding of psychophysiological states. Devices and software designed to receive and filter physiological waveforms, identify features of interest, calculate relevant metrics based on those features, and present information on the underlying physiological status back to the user form the backbone of real-time biofeedback approaches [10,11]. These types of sensors and algorithms have been successfully implemented to train users to improve physical symptoms (i.e., vestibular imbalances [12]), mental symptoms (i.e., depressive states [13]), and a variety of other clinical symptoms.

Biofeedback approaches relying on the detection of cardiovascular changes are particularly prevalent given the prominent and relatively isolated amplitude of the R-peak appearing within ECG waveforms, which represents ventricular contraction of the heart [14]. Subsequently, sensors designed to monitor cardiovascular changes have contributed substantially to the understanding of autonomic nervous system (ANS) changes [15]. For these approaches to be successful, it is critical that the feedback loop underpinning them operates in real-time or near real-time. While the shortest duration for traditional HRV analysis of ECG waveforms was historically identified as five minutes [16], more nuanced methods are required in order to accomplish on-line analysis. Specifically, this calls for ultra-short-term analytical approaches to calculate upon incoming data, which has become increasingly utilized in recent years [17].

Further, to ensure accuracy in real-time calculations, consideration must be given to the nature of the calculations, including the physiological processes they represent. HRV metrics consist of time-domain, frequency-domain, and geometrical approaches and can broadly be categorized according to the branch of the ANS contributing to its measurement [7]. In particular, the high frequency (HF) band (0.15–0.40 Hz) reflects respiratory sinus arrythmia, which is vagally mediated and reflects parasympathetic dominance. The low frequency (LF) band (0.04–0.15 Hz), in contrast, has been demonstrated as a proxy for sympathetic dominance by some, and sympatho-vagal balance by others [16]. The LF/HF ratio thus reflects an input from both branches of the ANS and, given their respective oscillation frequencies and generally slower sympathetic responsiveness, requires a minimum time window of one minute of data to discriminate between high and low arousal states accurately [18], and to perform on-line processing with accuracies equivalent to a 5-min time window [19,20]. Additionally, previous work has suggested a relationship between the LF/HF ratio and emotional states such as frustration [21], mental stress [22], and cognitive workload [23].

Insights into the surgical team members’ mental state and changing cognitive workload can be extrapolated according to underlying physiological changes via non-invasive sensors [24]. Estimation of intra-operative cognitive workload in particular is most often approached through HRV acquisition and analysis [25]. Previous work has demonstrated the sensitivity of capturing intra-operative HRV from multiple team members, analyzed according to surgical phase [26] and according to intra-operative events [27].

In addition to integrating data representing the progress of the surgical procedure and patient data, monitoring clinicians’ mental workload state may provide a more comprehensive snapshot of the COR context. In fact, clinician workload represents a unique data stream/input incorporated into the COR-ICE platform. The tenets of mental workload theory are the assumptions that individuals have a limited cognitive capacity, demands imposed and resources required vary according to the task at hand, and individuals differ in the degree of cognitive resources required to perform a given task [28]. Thus, monitoring individual operators during the course of a multi-task, multi-step procedure reflects personalized insights into cognitive efficiency. In the future, tying these insights to a process model of the specific procedure would allow immediate feedback based on both the current step in the process as well as predicted likely future states and actions. In surgery in particular, cognitive factors are the major contributors to human performance deficiencies and adverse events [29], necessitating a nuanced understanding of these cognitive workload changes over time.

In addition to deriving individual indicators of mental states using sensor technology, capturing and representing team cognitive load states is still facing a particularly compelling knowledge gap. Effective implementation of team cognitive load calculations has been demonstrated through off-line analysis in the domains of submarine navigation [30] and cardiac surgery [31,32], but its real-time implementation has received less attention.

The goal of this work is to develop an interoperable pipeline on an open-source platform to enable the real-time representation of a team cognitive load estimate derived from the simultaneous acquisition of ECG measurements from multiple team members.

## 2. Methods

We designed an architecture for real-time capture, analysis, and presentation of HRV data from multiple clinicians and implemented this architecture as an application and device interfaces on the OpenICE platform.

### 2.1. COR-ICE: OpenICE-Centered Context-Aware Operating Room

**Figure 1:**
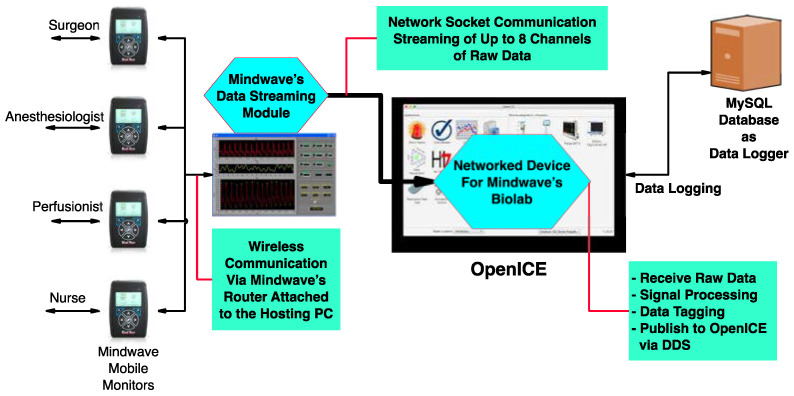
COR-ICE Architecture.

The COR-ICE architecture shown in Figure 1 brings together physiological data from multiple clinicians into an interoperable processing pipeline that supports individualized and team-based alerts and notifications. COR-ICE includes interfaces for the clinical team that provide a summary of the current data sources and HRV metrics for each clinician and a dashboard of team status.

OpenICE (OpenICE source code is freely available at github.com/mdpnp (accessed on 10 January 2023) and OpenICE.info) is intended as a distributed system centered on a single patient. One of the foundational insights in its design was that healthcare is already a distributed system; caregivers learn about the patient and their condition and share information with each other in both structured and un-structured ways as the information is needed and available. COR-ICE builds on this foundation to add multiple streams of clinician physiological data that can be associated with the clinical role and a specific patient, enabling alerts that could also take into account that patient’s status.

The design of OpenICE mirrors this architecture, with independent agents such as medical equipment interfaces and applications sharing data elements as needed via a publish/subscribe middleware. In a publish/subscribe communication pattern, data producers publish the produced data to different pre-defined topics, while data consumers who are interested in using the produced data for their tasks subscribe to these topics. In this pattern, topics can be considered as buffers from which data consumers can receive the latest data produced by data producers. We have found that these publish/subscribe patterns map well onto many clinical applications and that building on existing and familiar communication patterns enhances explainability; the way data moves around makes sense to many clinicians because it matches how they are used to working. OpenICE shares goals with other medical device interoperability projects such as the 11073-SDC (Service-oriented Device Connectivity) family of standards developed by the OR.net organization [33,34].

OpenICE emphasizes bedside networks and the capacity for closed-loop control based on requirements from specific clinical scenarios, while 11073-SDC has an emphasis on linking devices to enable data sharing using established web-services protocols. Both groups have complementary approaches and share the goals of improving the safety and quality of clinical care.

The COR-ICE architecture shown in Figure 1 supports connecting a wide variety of sensors and data sources. As many sources of contextual data are available for use in OR, COR-ICE provides a generalized, standards-based terminology set and means to capture and share data from sensors. The ICE standard explicitly includes the healthcare providers (HCP), connections to non-medical equipment in the room; tracking sound/noise levels and equipment state provides important contextual cues [35]. This aligns with the ultimate goal of this work—supporting the cardiac surgical team with relevant contextual information [36].

A wide range of monitoring modalities are available. In previous work, we proposed to monitor HRV and map this psychophysiological measure to cognitive workload [26]; we are extending this in COR-ICE to additionally monitor clinician eye-tracking, electroencephalography (EEG), Near Infrared Spectroscopy (NIRS), heart rate (ECG), pulse oximetry, body temperature, blood pressure, electrodermal activity (EDA), and voice recognition.

In this work, we integrate with OpenICE portable monitors that the team members can wear that will stream ECG and other physiologic signals from all of the participants simultaneously. Preoperative domain knowledge, patient information, and staff profiles could also be securely made available to the COR-ICE from the hospital PACS (Picture Archiving and Communication System) through the COR-ICE external interface and HL7 FHIR (Fast Healthcare Interoperability Resources) interfaces, though this would require further system integration work.

Software applications for the cardiac OR mostly fall into two categories: (1) smart alarms and dashboards that monitor the output of one or several connected devices and create alarms and alerts based on the data they receive; and (2) closed-loop control applications that automatically control the care delivery by one device based on sensor data from one or more other devices. These software applications are safety critical and will usually need to be hosted close to the patient rather than on a server in a datacenter; this reduces the risks of network delay and accidental disconnection, simplifies patient identification and association, and allows direct feedback to the clinicians at the patient’s side. These software applications for the cardiac OR can be implemented as ‘apps’ hosted inside the ICE Manager in OpenICE that is deployed close to the patient.

### 2.2. Deriving Cognitive Load Estimates and Alerts from Heart-Rate Variability Metrics

Dias et al. [5] introduced a dashboard for visualizing cognitive load of multiple clinicians in a surgical team. The proposed dashboard used recorded data and a commercial HRV analysis program to do offline analysis and visualization. We build on this work by creating an open-source processing pipeline for real-time monitoring and alerting based on live streams of data from the surgical team members, where components of the pipeline can be easily changed and reordered to accommodate different formats and quality levels of the input ECG data streaming.

### 2.3. Processing Pipeline

**Figure 2:**
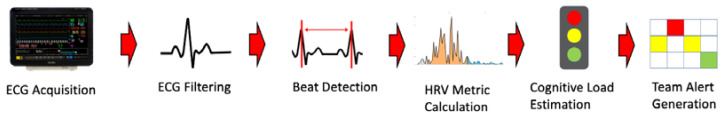
Stages of the Processing Pipeline.

The process of going from individual clinician ECG measurements to alert notifications based on the team’s cognitive load estimate includes six major stages as illustrated in Figure 2, including: (1) acquisition of ECG data; (2) ECG filtering to remove noise; (3) Beat detection and inter-beat interval (IBI) calculation; (4) HRV metric calculation; (5) cognitive load estimation; and (6) alert management. The inputs and outputs of each stage are enumerated in Table 1.

ECG acquisition starts with a medical device measuring electrical potential from multiple points on a person’s skin. This system was developed and tested with different devices: Mindware Mobile monitors, a Philips MX800 patient monitor, and a software simulated ECG device built into OpenICE. ECG waveforms at this stage include noise and artifacts from motion, poor electrode contact, and other causes.

ECG data originates as voltages measured using electrodes attached to the skin. Electrical potential differences between pairs of electrodes are represented by convention as virtual Leads. We used Lead II as the input for this processing pipeline. The Mindware Mobile, Philips MX800, and most other medical devices output data encoded in proprietary terminologies and communicate using proprietary protocols. The OpenICE equipment interfaces use these proprietary protocols to communicate with the devices and then republish the data in a standardized encoding. This encoding uses the ISO 11073-10101 [37] term “MDC_ECG_Lead_II” and represents the data as a series of voltage measurements with a sampling rate determined by the acquisition device. OpenICE bundles together 250 ms of waveform data into each waveform publication. The Mindware Mobile samples at 500 samples per second, so each MDC_ECG_Lead_II waveform publication would include 125 samples and a timestamp for the first sample in the array. This format is used between several of the stages of the processing pipeline and also in other applications, allowing easy use of other ECG acquisition devices and easy replacement or reordering of processing pipeline stages.

The filtering stage takes a standardized ECG waveform as input, applies signal processing to remove noise and artifacts, and outputs the ‘cleaned-up’ ECG as another standardized ECG waveform. We followed the approach of Chen et. al. [8] and designed our prototype application with three stages of filtering: wavelet denoising, linear high-pass filter, and non-linear low-pass filter. Any number of filtering stages could be applied here; each consumes and produces an ECG waveform so they can be combined in any desired order. Related work in de-noising and classifying individual beats [38,39,40] could plug in at this stage to reduce the effects of missed beats and noise on the downstream calculations of inter-beat intervals.

Accurate beat detection is critically important for the HRV analysis. Our implementation uses an adaptive threshold algorithm that continuously monitors the ECG signal to determine the highest voltage values (corresponding to R-wave peaks) over the last 10 s, then sets a slightly lower threshold value. When the input waveform exceeds this threshold, the algorithm watches for a change in sign—when the voltage switches from increasing to decreasing—that marks the peak of the R-wave. The time of this peak is the time of a heartbeat, and the time interval between this and next peaks is the R-R interval or inter-beat interval. Much related research in beat detection, for instance [41], could be leveraged here to improve performance.

The ECG sampling rate must be high enough to allow peak detection with minimal errors. Errors in the timing of R-waves will directly affect the accuracy of inter-beat intervals and all downstream metrics. Sampling rate relates to the accuracy of the timing measurements because the R-wave peak will fall within one to two sample periods. The interval depends on the time of two beats, so each interval has an error of ±2 sample periods. If the ECG is sampled at 100 Hz, each sample covers a 10 ms time period and the errors become a similar magnitude to the beat-to-beat variability being measured. This prototype application requires a sample rate of 500 measurements per second or higher to reduce the magnitude of timing measurement errors.

The sequence of IBIs is the basic input to the HRV metric calculations. There are many HRV metrics [7]; previous work [5] has found that RMSSD and LF/HF ratio are the most relevant for the cognitive load estimation stage that will use these metrics as input.

The modular architecture of the processing pipeline allows for easily providing additional inputs at any stage. Some devices, for instance the Polar H10 monitor [42], directly output and stream IBI sequences in real-time. Feeding these IBI sequences into the pipeline replaces the Acquisition, Filtering, and Beat Detection stages with the H10′s built-in algorithms. This has advantages for reducing the complexity of the system at the expense of also reducing flexibility—it is not possible, for instance, to change how the H10 filters ECGs before beat detection.

Our COR-ICE prototype implementation allows playback of recorded IBI sequences from Polar or processed output of programs such as Kubios that take ECG records as input, perform highly-configurable processing, and output IBI sequences. Analysis programs such as Kubios are useful tools for offline analysis but do not generally support real-time streaming or alert generation.

The six stages of our data pipeline are modularized and interoperable, meaning that their inputs and outputs of each stage are defined in a standardized terminology and the OpenICE platform provides a standardized means for communicating the data elements between stages or applications.

## 3. Results

We have built a prototype implementation of COR-ICE, an interoperable system for capturing and real-time analysis of multiple streams of ECG data and performed pre-clinical bench testing to verify that it meets key design metrics. It should be noted that this prototype does not intend to validate specific HRV metric calculations or cognitive load estimation algorithms. Rather, it intends to verify the system architecture, particularly the data pipeline described above, for future integration with and application in the context-aware OR to support clinical research on surgical process monitoring and modeling.

### System Implementation

Our COR-ICE prototype is implemented as an app on the OpenICE platform. As such, it inherits many capabilities and interfaces from the platform, including the real-time data bus and equipment interfaces. Figure 1 shows the main components of this app.

Multiple Mindware Mobile monitors are connected via a dedicated WIFI network to a computer running Mindware’s Biolabs software. The Mindware Data Streaming Module is used to collect and relay one channel of ECG data per monitor as a data stream over ethernet to an OpenICE equipment interface. The equipment interface receives the data stream, associates each ECG waveform with a unique device identifier, and publishes the data as an MDC_ECG_LEAD_II waveform on the DDS data bus. The data streaming module supports eight simultaneous waveform streams. The monitoring app is designed to support simultaneous monitoring of up to four members of the surgical team, but there is no fundamental limit preventing expansion to monitoring up to eight people with a single data streaming module or multiples of eight people with multiple Biolabs systems.

The COR-ICE app subscribes to these waveform data streams, calculates the HRV metrics, and creates a real-time display of metrics and cognitive load estimates. Data, including the calculated metrics and waveforms, is also recorded by the OpenICE’s built-in data logger (complying with the AAMI 2700-2-1: ICE Forensic Data Logging Standard [43]) with a coordinated timestamp to support future analysis and research.

**Figure 3:**
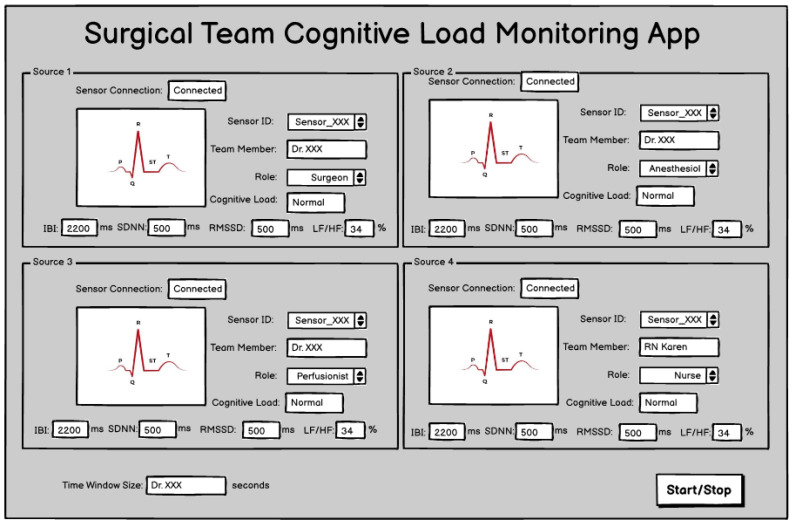
Application Design Wireframe.

**Figure 4:**
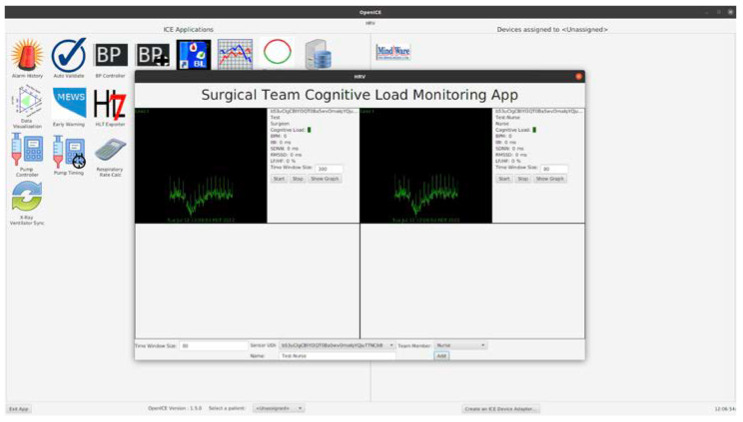
COR-ICE HRV Analysis App UI.

One challenge in developing this app is that the ICE architecture has primarily been used to host applications supporting the care of a single patient. While there is still a single patient in the cardiac operating theatre, the system must now track physiologic data from multiple individuals. This is handled in the HRV Analysis app by providing a user interface (shown in Figure 3 and Figure 4) with means for the user to associate ECG waveforms with a clinical role and, optionally, clinician name. In this implementation, the team roles are “Surgeon”, “Anesthesiologist”, “Perfusionist”, and “Nurse”.

Much of the complexity of the data processing pipeline is not exposed on the user interface. The user can specify the time window over which the HRV metric calculations are made, ranging from 10 s to 300 s. The average heart rate over this interval, SDNN, RMSSD, and LF/HF ratio over this time window are displayed for each clinician and the values are updated once per second.

The last layer of interpretation is estimating clinician cognitive load from the HRV metrics. The app will generate some threshold-based alerts from the HRV and/or Cognitive Load metrics. The cognitive load estimation and alerts are still under development. We plan to map terciles of LF/HF ratio to low/medium/high cognitive load estimates and display a ‘traffic light’ style section for each clinician, illustrated in Figure 4 as a small green box. For team alerts, we plan to assign a score of 1 to 3 to each tercile and set a team alarm under two conditions: if the sum of the four scores is ≥7 OR if two team members are in the red.

Validation that the cognitive load measurements accurately reflect the clinician’s actual cognitive load and that alerts are triggered at correct times will require running the app against recorded data sets where events and cognitive load levels are manually annotated. We are in the process of collecting such a data set and will pursue validation of the cognitive load estimation algorithm as future work. One key benefit of our approach is that any algorithm for cognitive load estimation based on ECG signals or sequences of HRV metrics can easily plug into other pipeline stages, allowing for quick testing and comparison of algorithms without needing to recreate data collection and filtering stages.

## 4. Conclusions

We have presented an overview of the COR-ICE architecture, an example scenario of the type of applications we are planning to address, and the data sources, including surgical team monitoring, that drive the awareness of context. We believe all of these elements are necessary to build a context-aware operating room for cardiac surgery.

Beyond the technical ‘bricks and mortar’ that make up the COR-ICE, we have a shared vision of how applications in a context-aware operating room will need to inter- act with the surgical team. Current medical devices and clinical applications typically trigger alarms and alerts based on a single monitored variable such as heart rate or blood pressure. Monitors and decision support systems that lack clinical context create non-actionable nuisance alarms that are not clinically relevant and cause distractions during the most difficult moments of a surgery. In contrast, we believe COR-ICE applications will be able to interact with the surgical team more like another team member. Communication between team members takes into account where they are in the surgical workflow, how busy or mentally occupied each team member is, and knowledge of who needs to know what to respond to expected and unexpected occurrences. By integrating contextual cues and team member state into a unified process model, COR-ICE applications will be able to emulate some of these behaviors to provide context-aware information to improve the safety and quality of surgical interventions.

This novel work demonstrates for the first time the feasibility of acquiring multiple ECG waveforms, applying an interoperable pipeline capable of filtering the ECGs, identifying IBIs, calculating HRV metrics, estimating team cognitive load states, and generating alerts in response. The real-time, multi-agent nature of this open-source platform advances the current state of sensor-derived signal analysis, with implications for providing cognitive support to teams operating in complex environments.

In the future, we envision further extensions to COR-ICE that will enable recording, in a common terminology and time-base, data from a rich variety of inputs including RGB-D cameras, real-time location systems, posture, gesture, instrument, and eye motion tracking, as well as object recognition, action detection, and detection of person-object interactions. Together with the current capability to stream and record vital signs and other information from the patient and clinical team, this will support integration with the workflow and process modeling to add awareness of the current status and stage of the surgical procedure.

Though preliminary, the demonstration of this pathway is unique in its utilization of data acquired in the naturalistic and complex environment of the cardiac operating room. Subsequent efforts will target validating the team cognitive load estimates derived algorithmically by observing event logs, team non-technical skills, and self-reports of stress and cognitive workload corresponding to the associated times. Excessive team cognitive load estimates will be validated against audio/video data capturing the procedures included in this analysis.

## Figures and Tables

**Table 1:** Processing Pipeline Stages, Inputs, and Outputs.

Stage	Input	Output
ECG Acquisition	Physiologic measurement	Standardized ECG waveform
ECG Filtering	Standardized ECG waveform	Standardized ECG waveform
Beat Detection	Standardized ECG waveform	IBI Series
Metric Calculation	IBI Series	HRV Metrics Series
CL Estimation	HRV Metrics Series	CL Estimate Series
Alerting	CL Estimate Series	Team CL Alerts

## Data Availability

Not applicable.

## References

[1] Wahr J.A., Prager R.L., Abernathy J., Martinez E.A., Salas E., Seifert P.C., Groom R.C., Spiess B.D., Searles B.E., Sundt T.M. (2013). Patient safety in the cardiac operat ing room: Human factors and teamwork. Circulation.

[2] Maier-Hein L., Vedula S.S., Speidel S., Navab N., Kikinis R., Park A., Eisenmann M., Feussner H., Forestier G., Giannarou S. (2017). Surgical data science for next-generation interventions. Nat. Biomed. Eng..

[3] Arney D., Rance G., Rithy S., Goldman J.M., Zenati M.A. (2018). A Novel Interoperable Safety System for Improved Coordination and Communication in Cardiac Surgery. OR 2.0 Context Aware Oper Theaters.

[4] Zenati M.A., Kennedy-Metz L., Dias R.D. (2020). Cognitive engineering to im- prove patient safety and outcomes in cardiothoracic surgery. Semin. Thorac. Cardiovasc. Surg..

[5] Dias R.D., Conboy H.M., Gabany J.M., Clarck L.A., Osterwei L.J., Avrunin G.S., Arney D., Goldman J.M., Riccardi G., Yule S.J. (2018). Development of an Interactive Dashboard to Analyze Cognitive Workload of Surgical Teams During Complex Procedural Care. IEEE Int. Interdiscip. Conf. Cogn. Methods Situat. Aware Decis. Support..

[6] (2019). AAMI American National Standard ANSI/ AAMI 2700-1:2019 Medical Devices and Medical Systems—Essential Safety and Performance Requirements for Equipment Comprising the Patient-Centric Integrated Clinical Environment (ICE)—Part 1: General Requirements and Conceptual Model.

[7] Shaffer F., Ginsberg J.P. (2017). An Overview of Heart Rate Variability Metrics and Norms. Front. Public Health.

[8] Szi-Wen C., Hsiao-Chen C., Hsiao-Lung C. (2006). A real-time QRS detection method based on moving-averaging incorporating with wavelet denoising. Comput. Methods Programs Biomed..

[9] Cutmore T.R.H., James D.A. (2007). Sensors and sensor systems for psychophysiological monitoring: A review of current trends. J. Psychophysiol..

[10] Schwartz M.S. (2010). A new improved universally accepted official definition of biofeedback: Where did it come from? Why? Who did it? Who is it for? What’s next?. Biofeedback.

[11] Vasilyev V., Borisov V., Syskov A. Biofeedback Methodology: A Narrative Review. Proceedings of the 2019 International Multi-Conference on Engineering, Computer and Information Sciences (SIBIRCON).

[12] Bowman T., Gervasoni E., Arienti C., Lazzarini S.G., Negrini S., Crea S., Cattaneo D., Carrozza M.C. (2021). Wearable devices for biofeedback rehabilitation: A systematic review and meta-analysis to design application rules and estimate the effectiveness on balance and gait outcomes in neurological diseases. Sensors.

[13] Melnikov M.Y. (2021). The Current Evidence Levels for Biofeedback and Neurofeedback Interventions in Treating Depression: A Narrative Review. Neural Plast..

[14] Shaffer F., McCraty R., Zerr C.L. (2014). A healthy heart is not a metronome: An integrative review of the heart’s anatomy and heart rate variability. Front. Psychol..

[15] Choi J., Gutierrez-Osuna R. Using Heart Rate Monitors to Detect Mental Stress. Proceedings of the 2009 Sixth International Workshop on Wearable and Implantable Body Sensor Networks.

[16] Task Force of the European Society of Cardiology and the North American Society of Pacing and Electrophysiology (1996). Heart rate variability: Standards of measurement, physiological interpretation and clinical use. Eur. Heart J..

[17] Shaffer F., Shearman S., Meehan Z.M. (2017). The Promise of Ultra-Short-Term (UST) Heart Rate Variability Measurements. Biofeedback.

[18] Salahuddin L., Cho J., Jeong M.G., Kim D. Ultra Short Term Analysis of Heart Rate Variability for Monitoring Mental Stress in Mobile Settings. Proceedings of the 29th Annual International Conference of the IEEE Engineering in Medicine and Biology Society.

[19] Baek H.J., Cho C.-H., Cho J., Woo J.-M. (2015). Reliability of Ultra-Short-Term Analysis as a Surrogate of Standard 5-Min Analysis of Heart Rate Variability. Telemed. E-Health.

[20] Schaaff K., Adam M.T.P. Measuring emotional arousal for online applications: Evaluation of ultra-short term heart rate variability measures. Proceedings of the 2013 Humaine Association Conference on Affective Computing and Intelligent Interaction.

[21] Kennedy-Metz L.R., Bizzego A., Dias R.D., Zenati M.A., Furlanello C. Autonomic Activity and Surgical Flow Disruptions in Healthcare Providers during Cardiac Surgery. Proceedings of the IEEE Conference on Cognitive and Computational Aspects of Situation Management.

[22] Ishaque S., Rueda A., Nguyen B., Khan N., Krishnan S. Physiological Signal Analysis and Classification of Stress from Virtual Reality Video Game. Proceedings of the 2020 42nd Annual International Conference of the IEEE Engineering in Medicine & Biology Society (EMBC).

[23] Böhm B., Rötting N., Schwenk W., Grebe S., Mansmann U. (2001). A prospective randomized trial on heart rate variability of the surgical team during laparoscopic and conventional sigmoid resection. Arch. Surg..

[24] Zhang H., Zhu Y., Maniyeri J., Guan C. Detection of variations in cognitive workload using multi-modality physiological sensors and a large margin unbiased regression machine. Proceedings of the 2014 36th Annual International Conference of the IEEE Engineering in Medicine and Biology Society.

[25] Daglius Dias R., Ngo-Howard M., Boskovski M., Zenati M., Yule S. (2018). Systematic review of measurement tools to assess surgeons’ intraoperative cognitive workload. Br. J. Surg..

[26] Kennedy-Metz L.R., Dias R.D., Conboy H.M., Nudel J., Stock E.M., Zenati M.A. Quantifying intraoperative team cognitive workload in complex surgical environments. Proceedings of the International Conference on Robotics and Automation.

[27] Zenati M.A., Leissner K., Zorca S., Kennedy-Metz L., Yule S.J., Dias R.D. (2019). First reported use of team cognitive workload for root cause analysis in cardiac surgery. Semin. Thorac. Cardiovasc. Surg..

[28] Babiloni F., Longo L., Leva M. (2019). Mental Workload Monitoring: New Perspectives from Neuroscience. Human Mental Workload: Models and Applications. H-WORKLOAD 2019. Communications in Computer and Information Science.

[29] Suliburk J., Buck Q., Pirko C., Massarweh N., Barshes N., Singh H., Rosengart T. (2019). Analysis of human performance deficiencies associated with surgical adverse events. JAMA Netw. Open.

[30] Stevens R., Galloway T., Wang P., Berka C., Tan V., Wohlgemuth T., Lamb J., Buckles R. (2013). Modeling the neurodynamic complexity of submarine navigation teams. Comput. Math. Organ. Theory.

[31] Dias R.D., Zenati M.A., Stevens R.H., Gabany J.M., Yule S.J. (2019). Physiological synchronization and entropy as measures of team cognitive load. J. Biomed. Inform..

[32] Kennedy-Metz L.R., Dias R.D., Stevens R.H., Yule S.J., Zenati M.A. (2020). Analysis of Mirrored Psychophysiological Change of Cardiac Surgery Team Members During Open Surgery. J. Surg. Educ..

[33] Kasparick M., Schmitz M., Golatowski F., Timmermann D. Dynamic remote control through service orchestration of point-of-care and surgical devices based on ieee 11073 sdc. Proceedings of the IEEE-NIH 2016 Special Topics Conference on Healthcare Innovations and Point-of-Care Technologies.

[34] Kasparick M., Schlichting S., Golatowski F., Timmermann D. New IEEE 11073 standards for interoperable, networked point-of-care medical devices. Proceedings of the 2015 37th annual international conference of the IEEE Engineering in Medicine and Biology Society (EMBC).

[35] Weininger S., Jaffe M.B., Robkin M., Rausch T., Arney D., Goldman J.M. (2016). The importance of state and context in safe interoperable medical systems. IEEE J. Transl. Eng. Health Med..

[36] Dias R.D., Yule S.J., Zenati M.A., Atallah S. (2021). Augmented Cognition in the Operating Room. Digital Surgery.

[37] (2020). Health informatics—Device interoperability—Part 10101: Point-of-care medical device communication—Nomenclature.

[38] Emina A., Subasi A. (2015). Effect of Multiscale PCA de-noising in ECG beat classification for diagnosis of cardiovascular diseases. Circuits Syst. Signal Process..

[39] Hammad M., Iliyasu A., Subasi A., Edmond H., El-Latif A.A. (2020). A Multi-tier Deep Learning Model for Arrhythmia Detection. IEEE Trans. Instrum. Measurement..

[40] Qaisar S.M., Subasi A. (2020). Cloud-based ECG Monitoring using Event-Driven ECG Acquisition and Machine Learning Techniques. Phys. Eng. Sci. Med..

[41] Patro K.K., Prakash A.J., Samantray S., Pławiak J., Tadeusiewicz R., Pławiak P. (2022). A Hybrid Approach of a Deep Learning Technique for Real–Time ECG Beat Detection. Int. J. Appl. Math. Comput. Sci..

[42] Schaffarczyk M., Rogers B., Reer R., Gronwald T. (2022). Validity of the Polar H10 Sensor for Heart Rate Variability Analysis during Resting State and Incremental Exercise in Recreational Men and Women. Sensors.

[43] (2022). Medical devices and medical systems-Essential Safety and Performance Requirements for Equipment Comprising the Patient-Centric Integrated Clinical Environment (ICE): Part 2-1: Particular Requirements for Forensic Data Logging.

